# Supplementary material for: Role and mechanism of NCAPD3 in promoting malignant behaviors in gastric cancer
Source: Front Pharmacol. 2024 Apr 22;15:1341039. doi: 10.3389/fphar.2024.1341039 (PMC11070777; doi:10.3389/fphar.2024.1341039)
Supplement: Supplementary file 11 [file DataSheet2.ZIP › GSEA/Oncogenic signature/my_analysis.Gsea.1599461962066/RPS14_DN.V1_DN.html]

Details for gene set RPS14\_DN.V1\_DN[GSEA]

|  || Dataset | filtered\_dataset.sample\_info.cls#WT\_versus\_NCAPD3\_MUT |
| Phenotype | sample\_info.cls#WT\_versus\_NCAPD3\_MUT |
| Upregulated in class | WT |
| GeneSet | RPS14\_DN.V1\_DN |
| Enrichment Score (ES) | 0.21015918 |
| Normalized Enrichment Score (NES) | 0.9769557 |
| Nominal p-value | 0.4974271 |
| FDR q-value | 0.6949261 |
| FWER p-Value | 1.0 |
Table: GSEA Results Summary

  

Fig 1: Enrichment plot: RPS14\_DN.V1\_DN      
 Profile of the Running ES Score & Positions of GeneSet Members on the Rank Ordered List

  

| SYMBOL | TITLE | RANK IN GENE LIST | RANK METRIC SCORE | RUNNING ES | CORE ENRICHMENT || 1 | 4288 | MKI67 | 1 | 1.341 | 0.0855 | Yes |
| 2 | 55149 | MTPAP | 15 | 1.051 | 0.1437 | Yes |
| 3 | 7052 | TGM2 | 17 | 1.045 | 0.2102 | Yes |
| 4 | 55240 | STEAP3 | 169 | 0.710 | 0.1467 | No |
| 5 | 54865 | GPATCH4 | 261 | 0.622 | 0.1210 | No |
| 6 | 27079 | RPUSD2 | 320 | 0.582 | 0.1165 | No |
| 7 | 1183 | CLCN4 | 373 | 0.551 | 0.1144 | No |
| 8 | 741 | ZNHIT2 | 453 | 0.503 | 0.0896 | No |
| 9 | 6470 | SHMT1 | 498 | 0.474 | 0.0883 | No |
| 10 | 2140 | EYA3 | 528 | 0.458 | 0.0968 | No |
| 11 | 27229 | TUBGCP4 | 556 | 0.442 | 0.1057 | No |
| 12 | 222658 | KCTD20 | 586 | 0.433 | 0.1126 | No |
| 13 | 23212 | RRS1 | 681 | 0.387 | 0.0696 | No |
| 14 | 6182 | MRPL12 | 729 | 0.359 | 0.0587 | No |
| 15 | 9238 | TBRG4 | 786 | 0.330 | 0.0395 | No |
| 16 | 51154 | MRTO4 | 794 | 0.327 | 0.0554 | No |
| 17 | 4285 | MIPEP | 815 | 0.307 | 0.0607 | No |
| 18 | 200315 | APOBEC3A | 866 | -0.278 | 0.0425 | No |
| 19 | 990 | CDC6 | 888 | -0.304 | 0.0469 | No |
| 20 | 79094 | CHAC1 | 949 | -0.367 | 0.0271 | No |
| 21 | 4194 | MDM4 | 1117 | -0.492 | -0.0619 | No |
| 22 | 8444 | DYRK3 | 1131 | -0.502 | -0.0390 | No |
| 23 | 29968 | PSAT1 | 1215 | -0.589 | -0.0611 | No |
| 24 | 440 | ASNS | 1232 | -0.609 | -0.0335 | No |
| 25 | 8501 | SLC43A1 | 1296 | -0.690 | -0.0347 | No |
| 26 | 3860 | KRT13 | 1367 | -0.847 | -0.0308 | No |
| 27 | 23650 | TRIM29 | 1384 | -0.951 | 0.0188 | No |
Table: GSEA details [plain text format]

  

Fig 2: RPS14\_DN.V1\_DN      
 Blue-Pink O' Gram in the Space of the Analyzed GeneSet

  

Fig 3: RPS14\_DN.V1\_DN: Random ES distribution      
 Gene set null distribution of ES for **RPS14\_DN.V1\_DN**
